# B and T cell response to SARS-CoV-2 vaccination in health care professionals with and without previous COVID-19

**DOI:** 10.1016/j.ebiom.2021.103539

**Published:** 2021-08-12

**Authors:** Andreas Zollner, Christina Watschinger, Annika Rössler, Maria R. Farcet, Agnes Penner, Vincent Böhm, Sophia J. Kiechl, Gerald Stampfel, Rainer Hintenberger, Herbert Tilg, Robert Koch, Marlies Antlanger, Thomas R. Kreil, Janine Kimpel, Alexander R. Moschen

**Affiliations:** a Christian Doppler Laboratory for Mucosal Immunology, Johannes Kepler University Linz, Linz, Austria; b Department of Medicine, Division of Internal Medicine 1 (Gastroenterology and Hepatology, Endocrinology and Metabolism), Medical University of Innsbruck, Innsbruck, Austria; c Department of Neurology, Medical University of Innsbruck, Innsbruck, Austria; d VASCage, Research Centre on Vascular Ageing and Stroke, Innsbruck, Austria; e Department of Hygiene, Microbiology and Public Health, Institute of Virology, Medical University of Innsbruck, Innsbruck Austria; f Global Pathogen Safety, Baxter AG (part of Takeda), Vienna, Austria; g Department of Internal Medicine 2 (Gastroenterology and Hepatology, Endocrinology and Metabolism, Nephrology, Rheumatology), Johannes Kepler University Linz, Linz, Austria

**Keywords:** Vaccination, Covid-19, SARS-CoV-2, T cell immunity, Pre-existing immunity, Humoral response

## Abstract

**Background:**

In recent months numerous health care professional acquired COVID-19 at the workplace resulting in significant shortages in medical and nursing staff. We investigated how prior COVID-19 affects SARS-CoV-2 vaccination and how such knowledge could facilitate frugal vaccination strategies.

**Methods:**

In a cohort of 41 healthcare professionals with (n=14) and without (n=27) previous SARS-CoV-2 infection, we assessed the immune status before, during and after vaccination with BNT162b2. The humoral immune response was assessed by receptor binding domain ELISA and different SARS-CoV-2 neutralisation assays using wildtype and pseudo-typed viruses. T cell immunity against SARS-CoV-2 surface and nucleocapsid peptides were studied using interferon-γ release assays and intracellular flow cytometry. Vaccine-related side effects were captured.

**Findings:**

Prior COVID-19 resulted in improved vaccine responses both in the B and T cell compartment. In vaccine recipients with prior COVID-19, the first vaccine dose induced high antibody concentrations comparable to seronegative vaccine recipients after two injections. This translated into more efficient neutralisation of virus particles, even more pronounced than expected from the RBD ELISA results. Furthermore, T cell responses were stronger in convalescents and particularly strong against the SARS-CoV-2 nucleocapsid protein.

**Interpretation:**

Herein, we corroborate recent findings suggesting that in convalescents a single vaccine dose is sufficient to boost adequate in vitro neutralisation of SARS-CoV-2 and therefore may be sufficient to induce adequate protection against severe COVID-19. New spike mutated virus variants render the highly conserved nucleocapsid protein – eliciting strong SARS-CoV-2 specific T cell immunity – an interesting additional vaccine target.

**Funding:**

Christian Doppler Research Association, Johannes Kepler University Linz

**Research in Context**

**Evidence before this study**

Vaccines against the coronavirus disease 2019 (COVID-19) are regarded the most promising strategy to contain the ongoing pandemic, caused by the severe acute respiratory syndrome coronavirus 2 (SARS-CoV-2). A few months after their emergency use authorization first insight into SARS-CoV-2 vaccine-induced immunity is emerging. Notably, a vast body of scientific evidence is available as preprints rather than peer-reviewed publications. Therefore, besides PubMed, preprint servers including MedRxiv, BioRxiv, and SSRN between Sept 1^st^, 2020 and June 5^th^, 2021 were screened for reports on SARS-CoV-2 vaccine response, using keywords including “SARS-CoV-2” “COVID-19” “vaccination”, “humoral response”, “T cells”, “pre-existing immunity”, and “secondary infection”. Knowledge about COVID-19 and SARS-CoV-2 vaccine immunology is changing rapidly, and weekly numerous new studies are published. We identified 14 peer reviewed original articles and research letters, which examined differences in vaccine response in convalescent and uninfected vaccine recipients (Table 1). Most of these studies describe differences in the humoral compartment of the immune system. Four studies additionally addressed cellular immunity. With one exception, all studies conclude that persons with a previous SARS-CoV-2 infection show an enhanced vaccination response, especially to the first vaccine dose.

**Table 1:** Peer-reviewed existing literature.

Study	Vaccine	Number of individuals [absolute (naïve + seropositive)]	Assay	Response	Comment
Krammer et al. 2021 [1]	BNT162b2 mRNA1273 (mixed)	109 (68 + 41)	ELISA	Spike IgG Side effects	only partially paired samples
Levi et al., 2021 [2]	BNT162b2	124 (67 + 57)	ELISA	Spike IgG	After 1^st^ vaccine dose
Jabal et al., 2021 [3]	BNT162b2	514 (497 + 17)	ELISA	Spike IgG	After 1^st^ vaccine dose
Saadat et al., 2021 [4]	BNT162b2 mRNA1273 (mixed)	59 (17 + 42)	ELISA	Spike IgG	After 1^st^ vaccine dose
Stamatatos et al., 2021 [5]	BNT162b2 mRNA1273 (mixed)	28 (13 + 15)	MNT ELISA Flow cytometry	Neutralisation of various virus variants RBD: IgA, IgG & IgM RBD specific IgG+ memory B cells Spike CD4+ T cells	
Reynolds et al., 2021 [6]	BNT162b2	51 (25+26)	ELISA MNT B and T cell ELISpot	Spike IgG Neutralising antibodies T cell response	After 1^st^ vaccine dose
Goel et al., 2021 [7]	mRNA-1273 BNT162b2 (mixed)	44 (33+11)	ELISA MNT ICFC B cell receptor sequencing	Spike IgG Antibody secreting cells SARS-CoV-2 specific T cells	4 timepoints
Mazzoni et al., 2021 [8]	BNT162b2	22 (11+11)	ELISA ICFC	Anti-spike: IgA, IgG, IgM CD4+ T-cells Memory B cells	After 1^st^ vaccine dose
Gobbi et al., 2021 [9]	BNT162b2	15 (9+6)	ELISA MNT	RBD/Spike IgG, IgM NP IgG	
Azzi et al., 2021 [10]	BNT162b2	65 (54+11)	ELISA	Anti-spike IgG	
Ebinger et al., 2021 [11]	BNT162b2	Before vaccination: 981 (903+78) After 1^st^ dose: 525 (490+35) After 2^nd^ dose: 239 (228+11)	ELISA	Anti-spike IgG	
Anichini et al., 2021 [12]	BNT162b2	99 (37+62)	ELISA MNT	Anti-spike IgG Neutralising antibodies	
Prendecki et al., 2021 [13]	BNT162b2	72 (56+16)	ELISA Elispot	Anti-spike & nucleocapsid IgG SARS-CoV-2 specific T cells	
Manisty et al., 2021 [14]	BNT162b2	51 (24+27)	ELISA	Anti-spike IgG	Only 1 timepoint (after vaccination)

ELISA=Enzyme linked immune sorbent assay. ICFC: intracellular flow cytometry. MNT=Micro neutralisation test. VSV=vesicular stomatitis virus. CyTOF= Cytometry by time of flight.

However, hardly any study reports longitudinal quantitative and qualitative antibody and T cell responses after administration of two doses of the BNT162b2 mRNA vaccine. Furthermore, so far, no study reported anti-SARS-CoV-2 neutralising capacity in international units following complete immunisation with BNT162b2.

**Added value of this study**

In this analysis of the BNT162b2 vaccine immunologic response, we corroborate recent findings that individuals with a prior SARS-CoV-2 infection exhibit a strong antibody response already after the first of two mRNA vaccine injections. This enhanced humoral immune response translates into a strongly improved virus neutralisation – more than would have been deduced from absolute anti-spike IgG concentrations. Furthermore, we demonstrate that vaccination of persons with prior COVID-19 results in increased numbers of SARS-CoV-2 specific T cells. Finally, our data suggests that the SARS-CoV-2 nucleocapsid protein represents a potent and promising T cell stimulant.

**Implications of all the available evidence**

Our findings are in support of the suggestion to develop a specific vaccine strategy for individuals with a history of COVID-19 optionally based on a simple immune monitoring before vaccination. In the context of spike mutated virus escape variants, we provide evidence that the nucleocapsid protein elicits strong SARS-CoV-2 specific T cell immunity, underscoring previous reports claiming that the nucleocapsid protein might feature an attractive additional target in future vaccine formulations.

Alt-text: Unlabelled box

## 1 Introduction

The coronavirus disease 2019 (COVID-19) pandemic represents an unprecedented crisis and threat to our healthcare systems due to limited capacities of standard and intensive care facilities. This situation is tightened by capacity shortfalls in healthcare worker resources driven by SARS-CoV-2 infections, quarantine regulations, and emotional and physical exhaustion [15]. Vaccines against SARS-CoV-2 are currently considered the most promising approach to face this global health threat.

The SARS-CoV-2 spike (S) protein, which includes the receptor binding domain (RBD), is key to cell entry for the virus and thus, was the primary target of all currently approved vaccines in Europe [16]. In addition to the spike protein, coronaviruses, including SARS-CoV-2, encode three other structural proteins, namely the envelope (E), membrane (M) and nucleocapsid (N) proteins, all of which evoke robust and detectable immune responses [17,18].

For the aforementioned reasons, health care workers are among the prioritised groups in most national vaccination programs. Rapid immunisation of this highly exposed group is of decisive importance to reduce virus transmission in hospitals and nursing facilities [19], and to preserve crucial human resources [20]. The early timepoint of vaccination, the well-known serostatus due to regular testing and the fact that a high percentage of health care professionals have already experienced an infection with COVID-19 [15], renders this group an interesting population to study immunologic effects of SARS-CoV-2 vaccines. By these means, academic supplementary programs paralleling the intended national vaccination programs may help to establish graduated vaccination recommendations particularly for people with pre-existing SARS-CoV-2 immunity [21]. As shown recently, most convalescents appear to have a protective humoral immune response for at least eight months [22], but various factors such as the viral load may influence quality and quantity of antibody formation [23].

To date, research focuses largely on antibody titres and their ability to neutralise virus particles. However, besides the humoral immune response, viral infections including COVID-19 typically shape virus-specific T cells [24]. Such T cell responses are more laborious to quantify than antibody concentrations and are therefore rarely used as screening and surrogate tools for detecting pre-existing coronavirus immunity. Nonetheless, T cell immunity is at least equally important in maintaining efficient immunity to B cell-mediated humoral responses and data on the original coronavirus SARS-CoV indicate that antibody titres tend to wane faster than specific T cell memory [25].

In this study, we carried out a detailed analysis of vaccine responses and side effects in health care professionals with and without previous exposure to SARS-CoV-2, to aquire new immunologic insights into both B cell and T cell immunity after vaccination against SARS-CoV-2.

## 2 Methods

### 2.1 Study participants

In this study, we compared the humoral and cellular immune response after routine application of the SARS-CoV-2 mRNA vaccine BNT162b2 from BioNTech/Pfizer in 41 staff members with (n=14) and without (n=27) a history of SARS-CoV-2 infection. The pre-vaccination COVID-19 exposure status was determined by a combination of medical history and serological testing for the presence of SARS-CoV-2-specific immunoglobulins before vaccination. All subjects classified as seropositive had a positive SARS-CoV-2 PCR test in their medical history and showed anti-SARS-CoV-2 potency in wildtype micro-neutralisation tests (MNT; Table 3). A detailed analysis of vaccine-related side effects after the second vaccine dose was carried out for all test subjects.

### 2.2 Quantification of humoral SARS-CoV-2 immunity

Antibodies were analysed in serum samples at the day of the first vaccination (day 0), on the day of the second vaccination (day 21) and four weeks after complete immunisation (day 49) using an RBD enzyme linked immunosorbent assay (ELISA) and functionality and quality of antibodies were determined by two previously described micro neutralisation tests (MNT) [26,27].

### 2.3 Anti-SARS-CoV-2 enzyme linked immunosorbent assays

SARS-CoV-2 spike and nucleocapsid protein specific immunoglobulins were quantified using an anti-SARS-CoV-2 QuantiVac ELISA (IgG) and an anti-SARS-CoV-2-NCP-ELISA (IgG) (both Euroimmun, Lübeck, Germany) according to manufacturer's instructions. All samples that were above the linear range at the recommended 1:101 dilution were further diluted 1:500 and 1:3000. According to the manufacturer's instructions ELISA antibody concentrations are reported in relative units (RU)/mL. Results in RU/mL correlate linearly with the first WHO international standard (NIBSC code: 20/136). Hence, sample values reported in RU/mL are convertible to international units (IU)/mL by multiplying with a factor of 3•2.

### 2.4 Micro neutralisation assays

In the SARS-CoV-2 micro neutralisation assay SARS-CoV-2 neutralising antibodies (nAb) titres were determined in human serum samples and serially diluted in 2-fold steps. Sample dilutions were mixed with virus stock at 10^3^ tissue culture infectious doses 50% per millilitre (TCID50/mL) SARS-CoV-2 (strain BavPat1/2020, kindly provided by C. Drosten and V. Corman, Charité Berlin, Germany) and incubated for 150 minutes, before titration on Vero cells (Cat. no. 84113001, European Collection of Authenticated Cell Cultures, Porton Down, Salisbury, UK) in 8-fold replicates per dilution. The virus-induced cytopathic effect was determined after 5-7 days of incubation. The reciprocal sample dilution resulting in 50% virus neutralisation (NT50) was determined using the Spearman-Kaerber formula, and the calculated neutralisation titre for 50% of the wells reported as 1:X. For further analyses, samples with a neutralisation titre below the detection limit were assigned a value of 0.5x the detection limit. The National Institute of Biological Standards and Control (NIBSC, Potters Bar, UK) WHO International Standard 20/136, for which a potency in international units has recently been assigned [28], was included in the study and the concentration of SARS-CoV-2 nAbs therefore reported in IU/mL. Testing was done using a fully ICH Q2R validated analytical method.

In the vesicular stomatitis (VSV) micro neutralisation test (VSV-MNT) titres of SARS-CoV-2 neutralising antibodies (nAb) were determined using a replication defective vesicular stomatitis virus (VSV) pseudo-typed with SARS-CoV-2 spike protein. Briefly, VSVΔG-GFP virus was produced on 293T cells stably expressing a C-terminally truncated version of SARS-CoV-2 spike (Wuhan isolate). Four-fold serial dilutions of heat-inactivated sera were pre-incubated with virus for 1h at 37°C and subsequently used to infect 293T-ACE2 cells seeded one day earlier. Approximately 16h after infection, plates were analysed using an ImmunoSpot S5 analyser. The number of GFP-positive cells was counted. The last plasma dilution that resulted in a 50% reduction of GFP positive cells compared to virus only wells was considered as 50% neutralisation titre. Results are reported as endpoint titres and titres of ≤1:4 were considered as negative, titres of ≥1:16 as positive.

### 2.5 Quantification of cellular SARS-CoV-2 immunity

The presence of SARS-CoV-2 specific T cells directed against the spike (S) and nucleocapsid (N) proteins were assessed with an interferon gamma release assay (IGRA) and flow cytometry four weeks after complete immunization (day 49). To specifically stimulate SARS-CoV-2 specific T cells, lithium heparin whole blood or isolated peripheral blood mononuclear cells (PBMC), were co-incubated with peptide pools (Miltenyi Biotech, Bergisch-Gladbach, Germany) consisting of 15-mer peptides with 11 amino acids overlap covering the entire sequence of the spike glycoprotein (pepS) and the complete sequence of the nucleocapsid phosphoprotein (pepN).

### 2.6 SARS-CoV-2 interferon gamma release assay

Cellular SARS-CoV-2 immunity was quantitatively analysed by a whole blood spike interferon−γ release assay (IGRA) and nucleocapsid IGRA respectively. Therefore blood was drawn into lithium heparin tubes and aliquoted into four 600ml whole blood conditions. The first aliquot was incubated with a SARS CoV-2 S-peptide cocktail, the second with an N-peptide cocktail, (PepTivator SARS-CoV-2 ProtS, S1, S+ & N, Miltenyi, Bergisch Gladbach, Germany). The third aliquot served as a negative control and was stimulated with the carrier liquid of the peptide pool (H_2_O). The fourth aliquot served as a positive control and was activated with 4 µg/mL phytohemagglutinin (PHA; Sigma, Missouri, US). After 24 hours on 37°C, the tubes were spun down at 2000 x g for 15 minutes and 200 µl aliquots were transferred into safelock tubes and stored at -80°C.

Concentrations of interferon gamma (IFN-γ) were measured using a human IFN-γ ELISA kit (BD OptEIA Set Human IFNg, BD Biosciences Pharmingen, New Jersey, US) according to the manufacturer's instructions. Negative control samples were diluted 1:5. SARS-CoV-2 peptide (pepS and pepN) co-incubated samples were diluted 1:5, 1:10, and 1:15. PHA stimulated samples were diluted 1:20 using dilution buffer.

### 2.7 Intracellular flow cytometry of SARS-CoV-2 specific T cells

To decipher subsets of the T cell compartment, SARS-CoV-2 specific T cells of seronegative (n=7) and seropositive (n=7) vaccine recipients were expanded and analysed by intracellular flow cytometry (ICFC).

First lithium heparin whole blood was collected and PBMCs were isolated using a Lymphoprep density gradient medium (Stemcell, Vancouver, Canada) according to the manufacturer's instructions. In brief 4 mL lithium heparin blood were diluted with 3 mL PBS, PBMCs were isolated by layering the 7mL on 4 mL Lymphoprep and subsequent density-gradient centrifugation for 30 minutes at 850 x g. PBMCs were collected from the interphase, washed twice, cryopreserved in heat- inactivated fetal calf serum (Sigma, Missouri, US) supplemented with 10% dimethylsulfoxid (Sigma, Missouri, US) and stored in liquid nitrogen until further use.

To expand SARS-CoV-2 reactive T cells, 2 × 10^5^ PBMCs in 200 µL of RPMI medium supplemented with 10% FCS were pulsed with 0•6 μg/mL spike (pepS, pepS1 and pepS+) or nucleocapsid (pepN) peptide pools in the presence of 10 U/mL interleukine-2 (IL-2). Cells were cultured with IL-2 only served as a negative control. After 60 hours cells were restimulated with or without 1 μg/mL pepS or pepN peptide pools. Cells stimulated with 4 μg/mL PHA served as positive controls. After combined surface (CD45, CD4, CD8, CD45RO, CD69) and intracellular cytokine staining (IFN-γ, TNFα, IL17A, granzymeB) specific T cell responses were acquired on a CytoFLEX Flow Cytometer (Beckman Coulter, California, US) and analysed with Flowjo v10.6 (Becton Dickinson, New Jersey, US). Antibodies and the respective suppliers are depicted in Table 2.

**Table 2:** Fluorochrome labelled antibodies and suppliers.

Reactivity	Fluorochrome	Supplier	RRID
CD4	Pe	Biolegend	AB_1937247
CD8	PerCP	Biolegend	AB_1575072
CD45	BV785	Biolegend	AB_2563128
CD45RO	PeDazzle	Biolegend	AB_2566542
CD69	BV605	Biolegend	AB_2562306l
IFN-γ	BV421	Biolegend	AB_2561398
TNFα	APC	Biolegend	AB_315264
IL17A	AF700	Biolegend	AB_2280255
GranzymeB	FITC	Biolegend	AB_2114575
Live/Dead	V510	Tonbo Biosciences	not available

(Biolegend, California, US; Tonbo Biosience, California, US).

### 2.8 Statistical analysis

Antibody concentrations and results obtained from IGRA are expressed as geometric means with 95% confidence interval (CI). After logarithmic transformation, differences in antibody concentrations between the seronegative and seropositive group and over the three time-points were analysed using a mixed-effects linear regression model with a random intercept for subjects and fixed effects for COVID-19 serostatus, sampling-time points, sex and the interaction between sex and COVID-19 serostatus. Differences between groups at each timepoint and antibody formation over time were further elucidated applying post hoc estimated marginal mean contrasts (R package: emmeans).

Categorical variables were analysed using the chi-squared test. Both in IGRA and flow cytometry, spike and nucleocapsid reactivity were calculated by subtracting the untreated response from that of the stimulation and differences between groups were calculated using the t-test. Correlations were determined using Pearson correlation analysis. Differences with p-values <0•05 were considered to be statistically significant. Analysis was conducted using Prism V9 (Graphpad, San Diego, US) and R version 4.1.0 (R Project for Statistical Computing, Vienna, Austria).

### 2.9 Ethics

All subjects received two vaccinations with BNT162b according to EMA approval. This study was approved by the ethics committee of the Johannes Kepler University Linz (EC-No. 1322/2020) and informed consent was obtained from all study subjects.

### 2.10 Role of the funding source

The sponsor of the study had no role in study design, data collection, data analysis, data interpretation, or writing of the report. All authors had full access to all the data in the study and the corresponding author had final responsibility for the decision to submit for publication.

## 3 Results

Despite stringent hygiene regulations including continuous wearing of FFP2 masks, safe distances and hand hygiene, 14 out of 41 staff members of the department were infected with SARS-CoV-2 between November 2020 and January 2021. No severe courses of COVID-19 occurred (Table 3). The serologic immune status matched with the reported SARS-CoV-2 status in 100% of cases. Accordingly, vaccine recipients were stratified into a group without (n=27) and with prior infetion with SARS-CoV-2 (n=14). Notably there was a sex imbalance in our cohort with more females (16/27) in the naïve for COVID-19 group and more males (9/14) in the status after COVID-19 group. However, this gender imbalance showed no statistically measurable influence on the antibody concentrations (mixed effects linear regression model). Clinical and demographic characteristics of this cohort are shown in Table 3.

**Table 3:** Characteristics of study population.

	Naïve for COVID-19 (Seronegative) (n=27)	Status after COVID-19 (Seropositive) (n=14)
Sex		
Female	16/27	5/14
Male	11/27	9/14
Age, years (IQR)	33•7 (29•8–43•5)	34•8 (28•5–43•2)
Vaccine=Bnt162b2 (%)	27/27	14/14
Ethnicity=Caucasian (%)	27/27	14/14
COVID-19 severity*		
No infection	27	NA
Asymptomatic	NA	0
Mild	NA	13
Moderate	NA	1
Severe, critical	NA	0
Virological parameters		
SARS-CoV-2 PCR positivity	0/27	14/14
SARS-CoV-2 RBD IgG positivity	0/27	14/14
SARS-CoV-2 neutralising potency	0/27	14/14
Days since PCR negativity	NA	25–61
Vaccine related side effects	18/27	13/14

⁎ COVID-19 severity is defined as follows• Mild, oxygen saturation always >90%. Moderate, decrease in oxygen saturation below 90%, but no oxygen supply necessary. Severe/critical, oxygen supplementation or high-flow oxygen or intubation.

After the second vaccination 18 out of 27 COVID-19 naïve participants experienced vaccination-associated symptoms, whereas 13 out of 14 individuals with recent COVID-19 reported side effects. These findings suggest that persons that have recovered from COVID-19 tend to experience more vaccination-related side effects (two-sided chi-squared test, p=0•142) (Table 3, appendix p3).

**Fig. 1:**
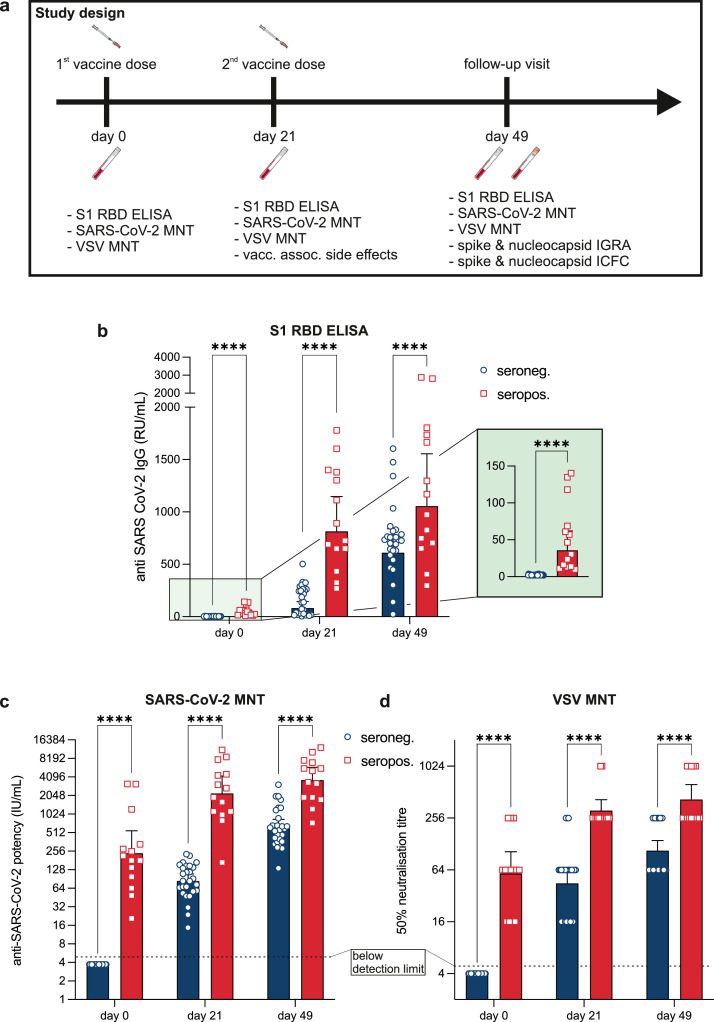
**a** Graphical illustration of the study design; **b** Assessment and comparison of SARS-CoV-2 S1 receptor binding domain (RBD) specific immunoglobulin G in seronegative individuals (blue; n=27) and individuals with pre-existing SARS-CoV-2 immunity (red; n=14) at the day of the first vaccine dose (day 0), at the day of the second vaccine dose (day 21) and four weeks after completed immunization (day 49). **c** Functional assessment of antibodies using a wildtype SARS-CoV-2 microneutralisation test. Detection limit was ≤4 IU/mL (dotted line). **d** Functional assessment of antibodies using spike pseudotyped vesicular stomatitis viruses. Titres ≤1:4 were counted as negative (dotted line). All symbols mark the reached 50% neutralising titre. Blue bars represent seronegative and red bars represent seropositive individuals. All bars represent geometric means and error bars 95% confidence intervals (not adjusted for multiple testing). Each symbol represents one test subject. Differences between subjects with and without history of COVID-19 were analysed at each time point by a mixed effects linear regression model adjusted for sex with posthoc estimated marginal mean contrasts. ****p<0•0001.

Antibody titres represent the hallmark of vaccine-induced immunity. Thus, we first compared serological responses between COVID-19-naïve study participants and individuals with a recent history of COVID-19. On day 0, no antibodies were identified in naïve subjects while antibodies were present in all vaccine recipients who had previously been in contact with SARS-CoV-2 (Table 4, Fig. 1b, appendix p4). In vaccine recipients with a history of COVID-19, the first vaccination induced a marked antibody production resulting in eight to nine-fold higher geometric means than in COVID-19 naïve subjects (Table 4, Fig. 1b). In both groups, the second injection further augmented the formation of RBD-specific immunoglobulin G (IgG). However, the antibody concentrations in convalescent subjects strongly exceeded those of COVID-19 naïve subjects (Table 4, Fig. 1b). Seroconversion was observed in 100% of subjects already on day 21 (Fig. 1b)**.**

**Table 4:** Anti-spike immunoglobulin G concentrations determined by enzyme-linked immunosorbent assay over time.

	Naïve for COVID-19 (Seronegative) (n=27)	Status after COVID-19 (Seropositive) (n=14)	p-value
Anti-Spike IgG (day 0), RU/mL	<8•0 (0)	31.8 (15•1–66•7)	<0•0001
Anti-Spike IgG (day 21), RU/mL	81•8 (46•6–143•4)	854•0 (611•5–1192•7)	<0•0001
Anti-Spike IgG (day 49), RU/mL	619•7 (454•9–844•3)	1061•8 (696•1–1619•5)	<0•0001

Data are geometric mean (95% CI). Statistical difference is calculated using a mixed effects linear regression model adjusted for sex with posthoc estimated marginal mean contrasts. IgG=immunoglobulin. RU=reference units.

To provide additional information on the functionality and quality of vaccination-induced antibodies we conducted two types of SARS-CoV-2 micro-neutralisation tests (MNT). First, we performed MNT using wildtype SARS-CoV-2. At day 0, subjects naïve for COVID-19 had no neutralising potency, while subjects with a history of COVID-19 showed a geometric mean neutralisation potency of 236•9 IU/mL (Table 5). Interestingly, despite higher absolute spike specific IgG in SARS-CoV-2 naïve subjects after the first vaccine dose (Table 4), the neutralising potency remained lower at 82•0 IU/mL (Table 5, Fig. 1c, appendix p5). The neutralising potency of both groups increased after the first and second immunisation, yet subjects previously exposed to COVID-19 consistently showed higher titres at all timepoints (Fig. 1c). Over all timepoints there was a strong correlation between the results from the MNT and RBD-ELISA assays (pearson r=0•91, p<0•0001).

**Table 5:** Neutralising potency determined by wildtype MNT over time.

	Naïve for COVID-19 (Seronegative) (n=27)	Status after COVID-19 (Seropositive) (n=14)	p-value
SARS-CoV-2 MNT50 (day 0), IU/mL	<4•0 (0)	236.9 (102•3–548•7)	<0•0001
SARS-CoV-2 MNT50 (day 21), IU/mL	82•0 (62•4–107•2)	2003•1 (1023•2–3921•4)	<0•0001
SARS-CoV-2 MNT50 (day 49), IU/mL	632•5 (475•9–840•5)	3626•9 (2234•0–5888•4)	<0•0001

Data are geometric mean (95% CI). Statistical difference is calculated using a mixed effects linear regression model adjusted for sex with posthoc estimated marginal mean contrasts. MNT50=microneutralisation titre 50%. IU=international units.

Secondly, we complemented neutralisation assays using spike-pseudotyped vesicular stomatitis viruses (VSV). Results obtained from the VSV-MNT correlated well with both data from the SARS-CoV-2 wildtype MNT (pearson r=0•93, p<0•0001) and IgG concentrations determined by RBD-ELISA (pearson r=0•87, p<0•0001). Again, at day 0, subjects naïve for COVID-19 had no neutralising activity (neutralising titres [NT]: ≤1:4) while subjects with a history of COVID-19 showed an average NT of 1:64. This was comparable with the titres that were found in the naïve for COVID-19 group 21 days after the first mRNA injection. At the same time serum from subjects with prior COVID-19 neutralised at a dilution of 1:256 or higher. In both groups, neutralising activity continued to rise until four weeks after the second dose. Notably, the neutralising capacity of initially seronegative subjects remained below the titres of convalescents (Fig. 1d).

**Fig. 2:**
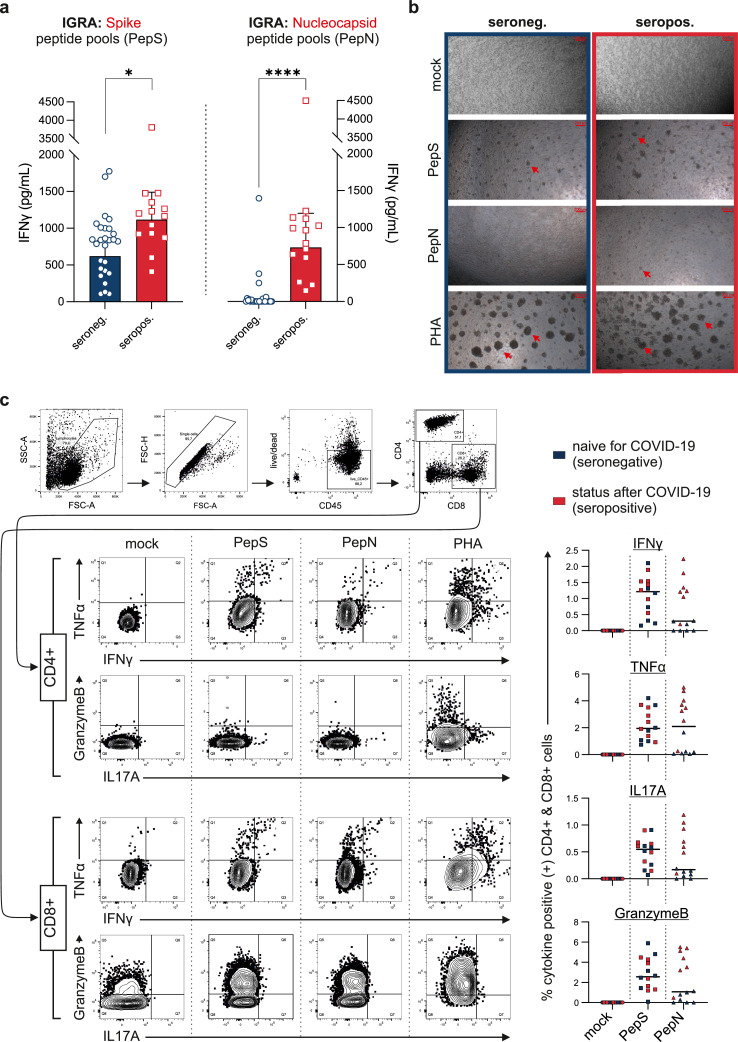
**a** At day 49 SARS-CoV-2 nucleocapsid and spike protein specific T cells were evaluated by interferon gamma release assays. Whole blood from all individuals (seronegative: n=27–blue; seropositive: n=14–red) was co-incubated with SARS-CoV-2 spike peptide pools (pepS, left side) and SARS-CoV-2 nucleocapsid peptide pools (pepN, right side). Bars represent the geometric means and error bars 95% confidence intervals. Symbols represents one test subject. Differences were analysed by t-test. *p<0•05; ****p<0•0001. **b** Representative microscopic pictures of at day 0 seronegative (left column) and seropositive (right column) peripheral blood mononuclear cells cultured in the presence of 1 µg/mL pepS and pepN (2^nd^ and 3^rd^ row). Mock and PHA treated cells served as negative and positive controls (1^st^ and 4^th^ row). Red arrows mark the formation of cell aggregates indicating proliferation and activation of T cells. Original magnification was 200-fold. **c** COVID-19 naïve subjects (n=7) and persons with a recent COVID-19 infection (n=7) were analysed by intracellular flow cytometry. On the left side, in an individual with prior COVID-19 a representative gating strategy and plots of intracellular IFN-γ, TNFα, IL17a and granzyme B in CD4+ T helper cells and CD8+ cytotoxic T cells are depicted. On the right side, the percentage of cytokine positive CD4+ cells and CD8+ cells are shown. Cytokine positivity was defined by subtracting the mock response from the pepN and pepS co-incubated samples. Blue symbols represent COVID-19 negative subjects, red symbols mark subjects with prior COVID-19.

Next, we examined cellular immunity and therefore carried out interferon-γ release assays (IGRA) utilising peptide pools that mapped the spike (S) and the nucleocapsid (N) proteins, respectively. As the S protein, which contains the receptor binding domain, is the vaccine target of BNT162b2, co-incubation of lithium heparin blood with spike peptide pools (pepS) resulted in marked IFN-γ responses in all test subjects. Notably, vaccine recipients with a prior COVID-19 history showed significantly stronger IGRA reactivities (1117•0 [95% CI 836•5–1491•0] pg/mL) indicating the presence of more SARS-CoV-2 spike protein-specific T cells compared to COVID-19 naïve vaccine recipients (620•6 [95% CI 460•2–836•9] pg/mL, t-test p<0•05; Fig. 2a). After the second vaccination, all test subjects demonstrated IFN-γ release upon pepS stimulation, whereas co-incubation with the nucleocapsid peptide pools (pepN) resulted almost exclusively in an IFN-γ release in subjects that had recovered from COVID-19 (1•1 [95% CI 0•3–4•0] pg/mL vs. 732•3 [95% CI 450•0–1192•0) pg/mL], t-test p<0•0001; Fig. 2a). Notably in subjects with prior SARS-CoV-2 infection the magnitude of pepS-IGRA response was strongly correlated with the pepN-IGRA response (pearson, r=0•86; p<0•0001; appendix p6).

To investigate a potential interdependence between cellular and humoral immunity, we correlated the magnitude of peptide stimulation induced T cell activity with the concentrations of RBD specific IgG and neutralising antibodies (appendix p6). Although we did not find a correlation between absolute RBD-IgG and spike-specific T cells (r_p_=0•08; p=0•62), the magnitude of spike IGRA T cell response exposed to be significantly associated with the absolute neutralising potency measured by SARS-CoV-2 MNT (r_p_=0•38; p<0•05; appendix p6).

In COVID-19 naïve recipients, pepN stimulation did not lead to any reaction in most cases. Interestingly, four individuals showed a positive signal upon pepN stimulation, even though the seronegative COVID-19 status was confirmed by absence of RBD specific antibodies and negative COVID-19 history. To examine this unexpected pepN IGRA response, we also tested for nucleocapsid specific antibodies at all timepoints. Notably, only one patient exhibited borderline anti-nucleocapsid IgGs.

To substantiate the data derived from the IGRA assays we performed combined surface and intracellular flow cytometry (ICFC) on unstimulated, specifically stimulated (pepN or pepS) or phytohemagglutinin (PHA) stimulated PBMC. During T cell expansion, co-incubation of cells with pepS, pepN and PHA resulted in the formation of cell aggregates, indicating cell activation and proliferation (Fig. 2b). While PHA stimulation, which leads to unspecific linking of T cell receptors, resulted in the formation of large cell aggregates, cell aggregates were smaller after pepS and pepN stimulation indicating the activation and proliferation of specific T cell clones. In line with the results from our IGRA assays, co-incubation with pepN resulted in the formation of T cell aggregates in subjects with previous COVID-19 only, while in the COVID-19 naïve group T cells were not affected by pepN (Fig. 2b).

After stimulation and incubation, cells were fixed and stained with a combination of surface lineage markers CD45, CD3, CD4, and CD8, cell activation markers CD45RO and CD69 and intracellular cytokines including IFN-γ, TNFα, IL-17A and granzyme B. Both CD4+ T helper cells and CD8+ cytotoxic T cells were activated by pepS in all participants; subjects with a history of COVID-19 also responded to pepN (Fig. 2c). Coincubation with SARS-CoV-2-specific peptides elicited IFN-γ+ T_H_1 and IL17A+ T_H_17 cells. Additionally, in cytotoxic CD8+ T cells stimulation with SARS-CoV-2 specific peptides resulted in an upregulation of intracellular granzyme B. Mathematical back calculations of flow cytometry data suggested that four weeks after the 2^nd^ vaccination between 0•07% and 6•7% of all T cells were specific for peptides of the spike protein. Again, the amount of intracellular cytokines of nucleocapsid treated cells from seronegative donors did not differ from mock treated cells.

## 4 Discussion

Here, we report new insights into our understanding of how vaccination against SARS-CoV-2 elicits immune memory in subjects with and without prior COVID-19. For that purpose we examined the cellular and humoral immune status before, in between and after immunisation with two doses of the BNT162b mRNA vaccine in a cohort of 41 health care professionals, of which 14 had pre-existing immunity to SARS-CoV-2.

Achieving a high vaccination coverage represents the most promising strategy against the current COVID-19 pandemic. In many countries the amount of supplied vaccine represents the most critical bottleneck to achieve this goal. To make the best possible use of the scarce vaccine supply, Krammer et al. suggested that one vaccine dose might be sufficient to induce adequate antibody titres in individuals with a prior history of COVID-19 [1]. In line with these findings, our ELISA results show that after a single vaccine dose antibody concentrations of convalescents exceeded those of COVID-19 naïve subjects by far. In the majority of cases after one vaccine dose RBD specific immunoglobulin G of convalescents even outnumbered those of naïve vaccine recipients after full immunization with two mRNA injections. Intriguingly, antibody concentrations induced by vaccination were 10-fold higher than titres induced by SARS-CoV-2 infection. This translated into an enhanced SARS-CoV-2 neutralising potency in convalescents in the microneutralisation tests (MNT) which was evident at all observation time points. Neutralising activity increased after vaccination in naïve vaccine recipients, yet to achieve a neutralising potency comparable to unvaccinated convalescents required full immunisation with two vaccine doses. At the end of our observation period, the neutralising potency in naïve subjects was only one sixth of that of convalescent vaccine recipients.

Our data are consistent with MNT data reported from the pivotal BNT162b2 phase III trial. Here, unvaccinated convalescents had neutralisation titres (NT) of 1:94 compared to an NT of 1:360 in naïve individuals following full vaccination [29]. Notably, this three-to-four-fold increase corresponds with our data where vaccination of naïve subjects resulted in a neutralising potency approximately threefold higher than in unvaccinated post-COVID-19 individuals. Due to the lack of an international standard at the time of the BNT162b2 phase III vaccine report, Pfizer-BioNTech was unable to report their neutralisation data in international units [29]. Here, we are the first to report MNT results post BNT162b2 vaccination in IU/mL and thus facilitating assay to assay comparisons for future studies. Using neutralisation data from 438 convalescents previously reported by us [26], we were able to further validate and confirm our data on natural infection versus vaccine induced neutralising potential.

The antigenic drift of SARS-CoV-2 has led to the emergence of several new escape variants, of which the delta variant appears to be probably the most worrisome at present [29]. Many of these spike mutations result in resistance to neutralisation by antibodies [30]. An accumulation of such mutations is moving the SARS-CoV-2 virus in a direction that could ultimately lead to further new variants escaping current prophylactic measures targeting the viral spike protein. Unbridled spread of the virus could eventually lead to further accumulation of critical mutations, with the ultimate consequence that we are constantly chasing new variants. Such considerations require that we best contain viral transmission and achieve an adequate immunity in the population. Reported cases of reinfection and infection after SARS-CoV-2 vaccination prompted comments advocating the need for an additional third booster dose for COVID-19 naïve vaccine recipients. Since in vitro neutralisation seems to be the best surrogate for immune protection [31], from our data it is plausible that one vaccine dose sufficiently induces protection in persons with prior COVID-19. It is still a remaining question how long this protection will last. Our findings emphasise the need for further studies to determine if, and if so, when people with a history of COVID-19 need another booster dose.

Besides antibody response, viral infections and vaccines also elicit virus-specific T cell response [32]. A recent study detected long lasting memory T cell immunity specific for the original SARS-CoV, even 17 years after the initial infection. Notably, these SARS-CoV-specific T cells were almost exclusively directed against the N protein [24]. This prompted us to expand our studies with assays for cellular immunity. This is often accomplished by methods such as flow cytometry or ELISPOT requiring isolation and expansion of primary immune cells [8], arguably distorting the *in vivo* situation and limiting the diagnostic value of cellular immunity. To overcome these problems, we established a standardised IGRA for the spike and the nucleocapsid proteins with a pre-defined incubation time. In accordance with the serostatus, pre-existing immunity was associated with increased cytokine concentrations in the pepS IGRA. These findings correspond well with the data recently published by Prendecki et al., who reported results from an spike ELIspot assay in which convalescent vaccine recipients demonstrated increased numbers of spot forming units after a single dose of BNT162b2 [13].

In the pepN IGRA all convalescent vaccine recipients displayed high IFN-γ concentrations. Although nucleocapsid specific T cells were not boosted by vaccination against the spike protein, in subjects with a history of COVID-19, pepN stimulation elicited a similar IFN-γ response as the pepS IGRA, indicating comparable numbers of nucleocapsid and spike specific T cells.

These findings suggest the SARS-CoV-2 nucleocapsid protein as a potent T-cell stimulus and warrants further research towards the development of an additional vaccination target. This is of particular importance as mounting evidence indicates that the spike protein is prone to immune escape as demonstrated in the South African virus variant B.1.351 or 501Y.V2 [33,34]. In contrast, the nucleocapsid gene appears more conserved and stable – another reason for considering the N protein, in addition to spike-related sequences, a promising future vaccine target [35,36]. The concept of adapting vaccines to stimulate T cells more effectively appears particularly interesting as two recent studies provide evidence that SARS-CoV-2 infected individuals typically generate T cells that target at least 15-20 different fragments of intracellular and surface coronavirus proteins and mostly do not target regions that were mutated in two recently discovered mutants [37,38]. In contrast to antibodies, which theoretically induce a “sterilising immunity” by blocking epitopes within the RBD [39]. T cell immunity arguably becomes relevant during the early infection, helping to contain coronaviruses leading to milder disease courses and to reduce the risk of contagion.

Noteworthy, the PepN IGRA identified three COVID-19-naïve vaccine recipients with nucleocapsid-reactive T cells. These individuals had no relevant antibody concentrations against both spike and nucleocapsid proteins. We favour two models that might explain these results. First, these pepN IGRA responses might represent a cross-reaction with one of the other six human pathogenic coronaviruses, most likely one of the four endemic common cold coronaviruses [40]. Second, as all subjects were in close contact to SARS-CoV-2 infected individuals at their workplace, it seems conceivable that exposure to very low concentrations of SARS-CoV-2 is insufficient to induce B cell response and symptomatic COVID-19, yet to elicit T cell memory.

Using flow cytometry, we showed that the SARS-CoV-2 vaccine not only induced a T_H_1-IFN-γ immune response but also triggered a T_H_17-IL17A immune response. The comparably small sample size used for the flow cytometry studies prevented us from dissecting differences in cytokine patterns between pepS and pepN stimulated cells. Arguably, our data support a concept where MHC-mediated presentation of virus peptides triggers antiviral T_H_1 and T_H_17 immune responses irrespective of the underlying peptides [41].

We provide new and detailed evidence that the SARS-CoV-2 serostatus strongly modulates vaccine immunology. Our data corroborate recent findings suggesting that already a single mRNA-based vaccine injection induces a robust immune response in convalescents. We were able to quantitatively compare spike and nucleocapsid reactive T cells and highlight that the N protein represents a surprisingly potent T cell stimulant. New spike mutated virus variants render the highly conserved N protein as an additional vaccine target of interest.

## Contributors

ARM, RH, HT, RK and AZ conceptualised and designed the study. ARM was the principal investigator and oversaw all aspects of the study. CW, AR, JK, MF, TRK and AZ carried out all experimental procedures. MF, TRK, ARM, SK and AZ analysed the data and prepared the tables and Fig.s. AZ, AP, RH, MA, GS, RK and VB were responsible for the data collection and CW, AZ and ARM verified the data. ARM, SK and AZ drafted the manuscript. All authors contributed to the findings, editing of the article, and approval of the final submitted version. All authors had full access to all data of the study and accept responsibility for the decision to submit for publication.

## Declaration of Competing Interest

TRK and MF are employees of Baxter AG, Vienna, Austria, now part of the Takeda group of companies and have Takeda stock interest. All other authors declare no competing interests.

## Appendix Supplementary materials

Image, application 1
