# Supplementary material for: B and T cell response to SARS-CoV-2 vaccination in health care professionals with and without previous COVID-19
Source: eBioMedicine. 2021 Aug 12;70:103539. doi: 10.1016/j.ebiom.2021.103539 (PMC8358275; doi:10.1016/j.ebiom.2021.103539)
Supplement: Supplementary file 1 [file mmc1.docx]

**Appendix**

**B and T cell response to SARS-CoV-2 vaccination in health care professionals with and without previous COVID-19**

**Authors**

Andreas Zollner, Christina Watschinger, Annika Rössler, Maria R Farcet, Agnes Penner, Vincent Böhm, Sophia J Kiechl, Gerald Stampfel, Rainer Hintenberger, Herbert Tilg, Robert Koch, Marlies Antlanger, Thomas R Kreil, Janine Kimpel & Alexander R Moschen

**Christian Doppler Laboratory for Mucosal Immunology, Johannes Kepler University Linz, Linz, Austria** (A Zollner MD, C Watschinger MSc, Prof AR Moschen); **Department of Medicine, Division of Internal Medicine 1 (Gastroenterology and Hepatology, Endocrinology and Metabolism), Medical University of Innsbruck, Innsbruck, Austria** (A Zollner MD, Prof Herbert Tilg, Prof R Koch, Prof AR Moschen); **Department of Neurology, Medical University of Innsbruck, Innsbruck, Austria** (SJ Kiechl MD); **VASCage, Research Centre on Vascular Ageing and Stroke, Innsbruck, Austria** (SJ Kiechl MD); **Department of Hygiene, Microbiology and Public Health, Institute of Virology, Medical University of Innsbruck, Innsbruck Austria** (A Rössler MSc, J Kimpel PhD); **Global Pathogen Safety, Baxter AG (part of Takeda), Vienna, Austria** (TR Kreil PhD, MR Farcet PhD) **and Department of Internal Medicine 2 (Gastroenterology and Hepatology, Endocrinology and Metabolism, Nephrology, Rheumatology), Johannes Kepler University Linz, Linz, Austria** (A Penner MD, V Böhm MD, G Stampfel MD, R Hintenberger MD, M Antlanger MD, Prof AR Moschen)

*Correspondence to:*

Prof. Alexander R Moschen MD PhD

Department of Internal Medicine

Christian Doppler Laboratory for Mucosal Immunology

Johannes Kepler University Linz

Krankenhausstr. 9, 4211 Linz, Austria

T: +43 5 7680 83 0

F: +43 5 7680 83 0

E: alexander.moschen@jku.at

Contents

[2 Supplementary Figures 3](#_Toc77957033)

[2.1 Supplementary Figure 1 3](#_Toc77957034)

[2.2 Supplementary Figure 2 4](#_Toc77957037)

[2.3 Supplementary Figure 3 5](#_Toc77957039)

[2.4 Supplementary Figure 4 6](#_Toc77957040)

# Supplementary Figures

## Supplementary Figure 1


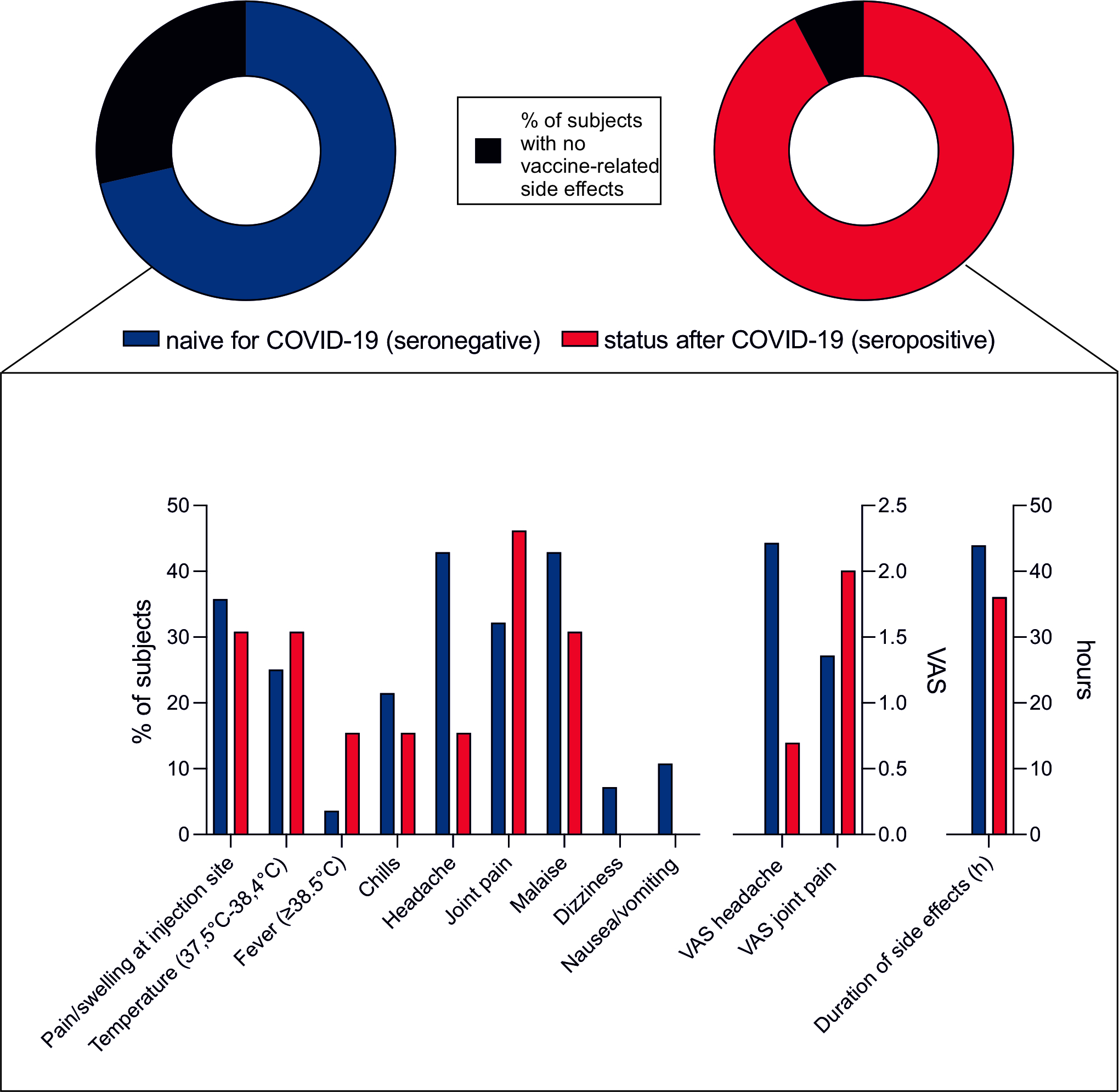


**Supplementary Figure 1:** *Vaccine-associated side effects experienced after the second mRNA vaccine injection (n=41, seronegative=28).* Bars represent the frequency of each symptom.

## Supplementary Figure 2


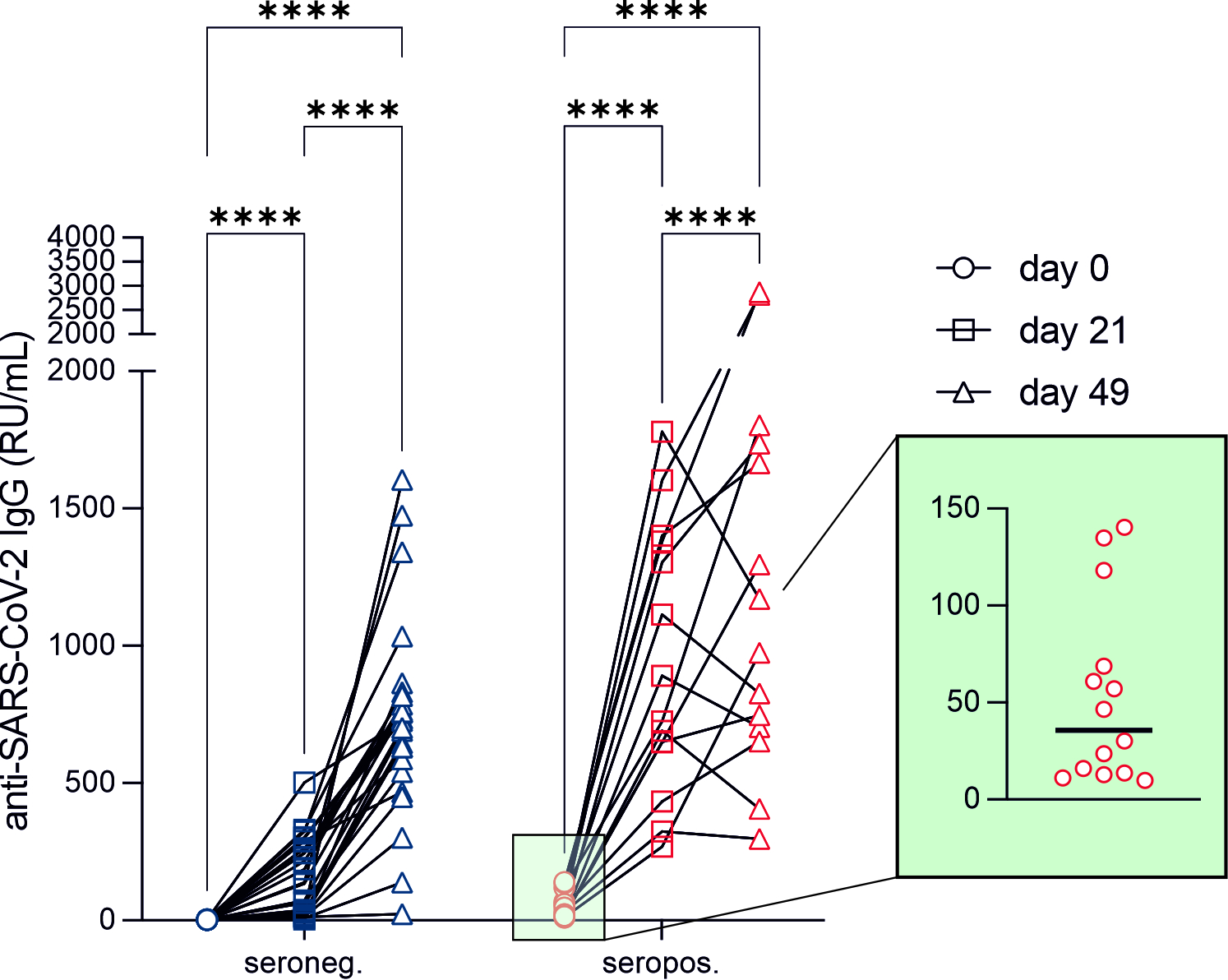


**Supplementary Figure 2:** *Assessment and comparison of SARS-CoV-2 S1 receptor binding domain specific IgG in seronegative individuals (blue; n=27) and individuals with pre-existing SARS-CoV-2 immunity (red; n=14) at the day of the first vaccine dose (day 0) at the day of the second vaccine dose (day 21) and four weeks after completed immunization (day 49). Each “circle-quadrat-triangle combination” connected with a line represents one test subject and shows antibody formation over time. Differences were analysed using a mixed effects linear regression model with posthoc estimated marginal mean contrasts. ****p<0*·*0001*

## Supplementary Figure 3


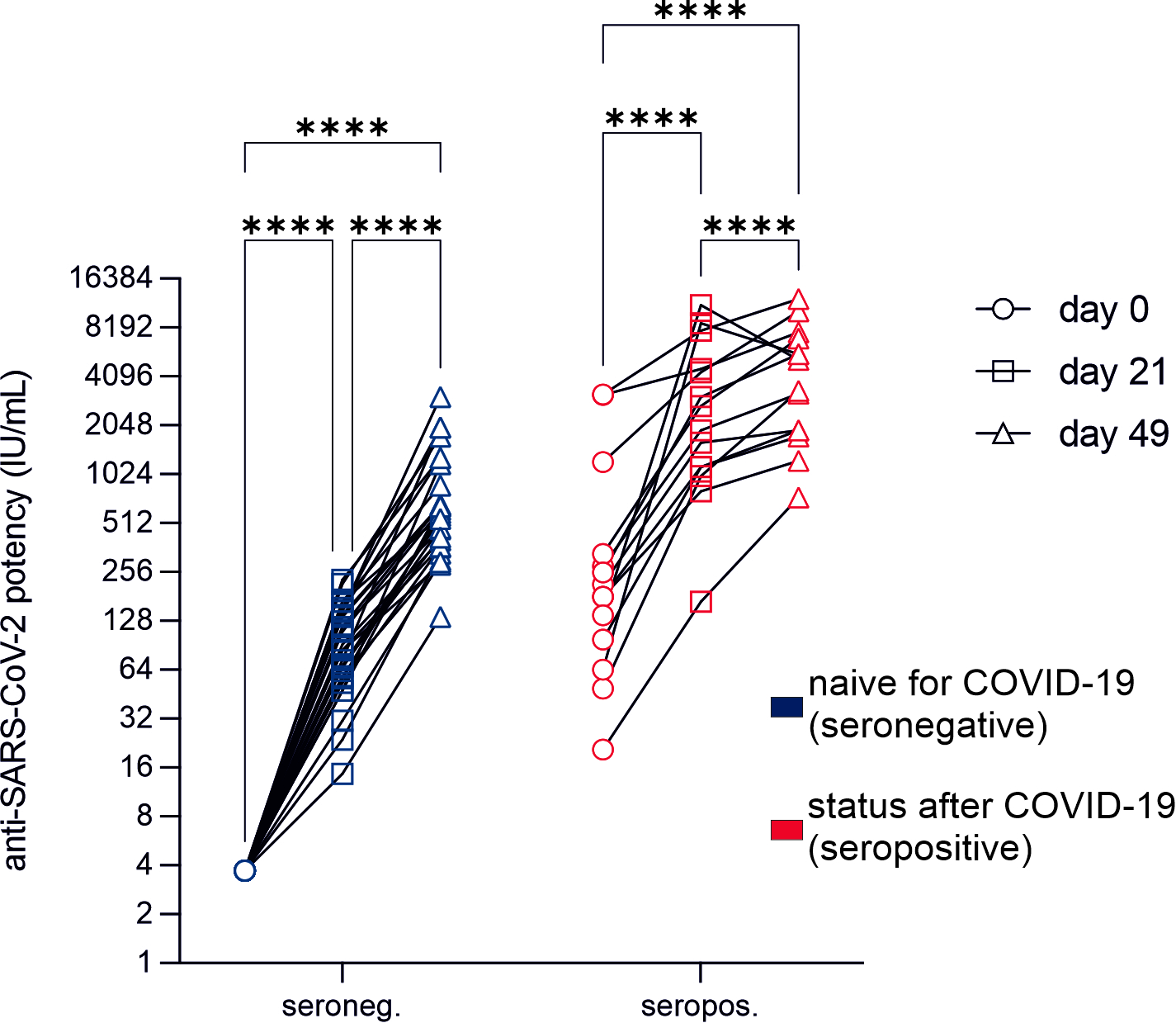


**Supplementary Figure 3:** *Assessment and comparison of SARS-CoV-2 neutralising potency in seronegative individuals (blue; n=27) and individuals with pre-existing SARS-CoV-2 immunity (red; n=14) at the day of the first vaccine dose (day 0) at the day of the second vaccine dose (day 21) and four weeks after completed immunization (day 49). Each “circle-quadrat-triangle combination” connected with a line represents one test subject and shows neutralising potency over time. Differences were analysed using a mixed effects linear regression model with posthoc estimated marginal mean contrasts. ****p<0*·*0001*

## Supplementary Figure 4


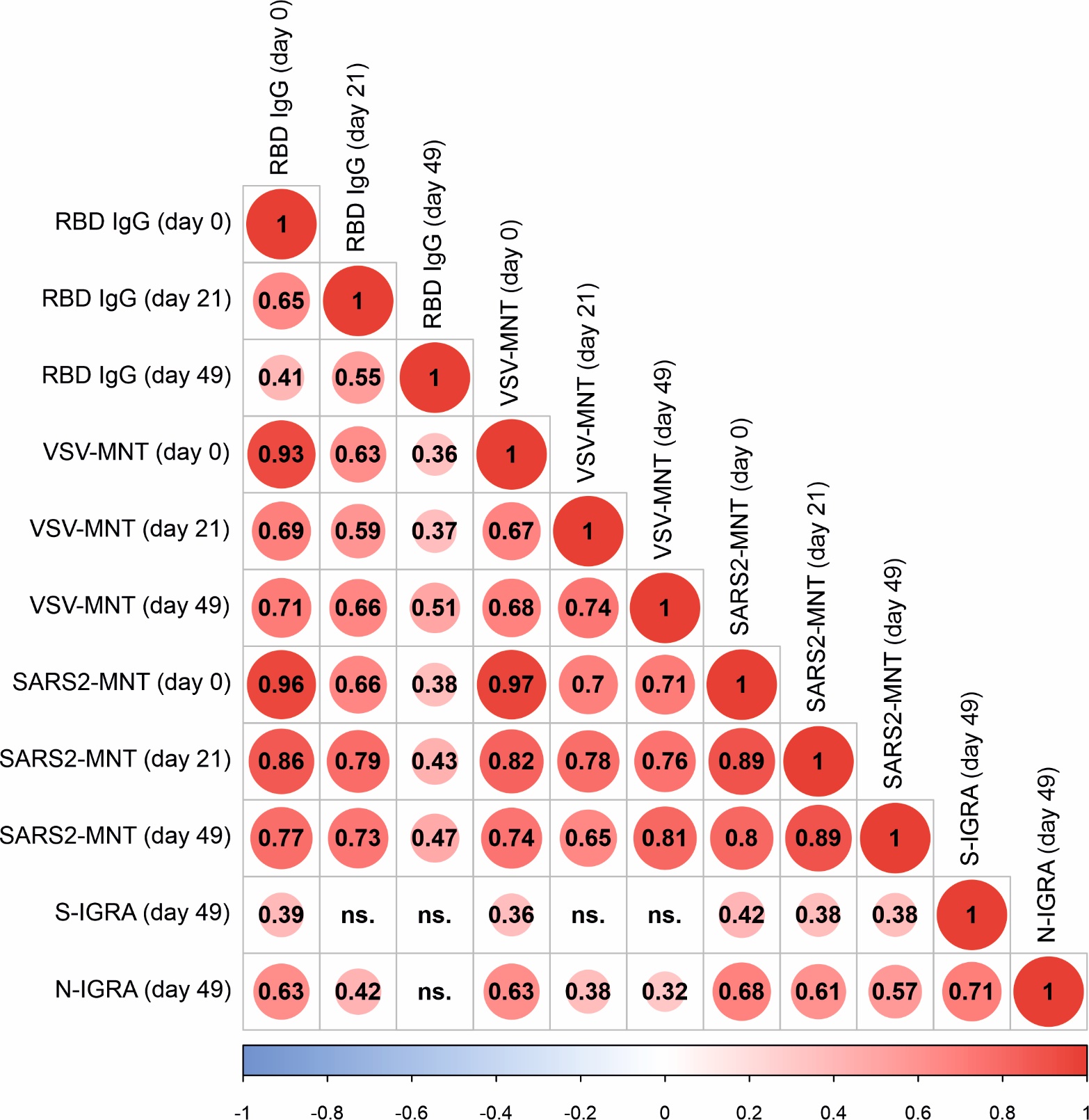


**Supplementary Figure 4:** *After logarithmic transformation of antibodies, correlation for all subjects was calculated using the Pearson correlation. Significant correlation coefficients are displayed.*
